# Supplementary material for: Transcriptomic Insights into Mechanisms of Early Seed Maturation in the Garden Pea (Pisum sativum L.)
Source: Cells. 2020 Mar 23;9(3):779. doi: 10.3390/cells9030779 (PMC7140803; doi:10.3390/cells9030779)

Zhewan−1(25) vs Zhon

Global Summary

%DE = 0.86

genes with fdr < 0.2 = 1747 (748 + / 999 −)

genes with fdr < 0.1 = 1630 (677 + / 953 −)

genes with fdr < 0.05 = 1486 (602 + / 884 −)

genes with fdr < 0.01 = 1217 (481 + / 736 −)

genes in genesets = 0

<FC> = 0

<t−score> = −11070279.81

<p−value> = 0

<fdr> = 0.14

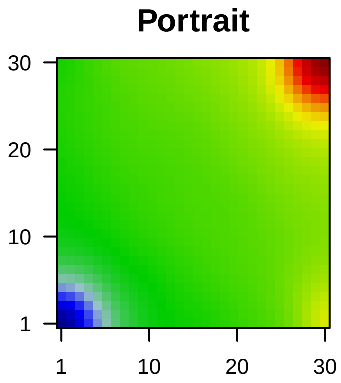

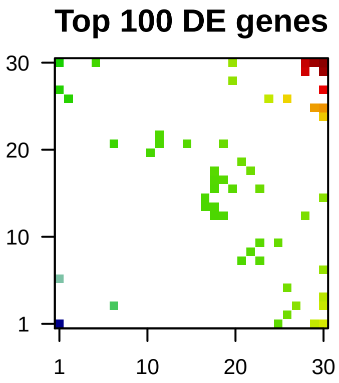

Global Genelist

Rank	ID	log(FC)	p−value	fdr	Metagene	Description
1	TRINITY_DN4	−10.05	0e+00	5e−10	1 x 1	
2	TRINITY_DN1	3.5	0e+00	5e−10	30 x 30	
3	TRINITY_DN1	0.02	2e−12	4e−09	15 x 21	
4	TRINITY_DN3	0.02	2e−11	4e−09	26 x 2	
5	TRINITY_DN1	0.02	3e−11	4e−08	18 x 17	
6	TRINITY_DN1	3.04	2e−10	4e−08	30 x 30	
7	TRINITY_DN1	0.08	4e−10	4e−08	21 x 19	
8	TRINITY_DN2	0.03	7e−10	4e−08	19 x 17	
9	TRINITY_DN1	0.05	7e−10	4e−08	20 x 28	
10	TRINITY_DN1	0.05	7e−10	4e−08	23 x 16	
11	TRINITY_DN2	0.12	7e−10	1e−06	27 x 3	
12	TRINITY_DN2	0.43	7e−09	1e−06	26 x 26	
13	TRINITY_DN2	3.06	7e−09	1e−06	30 x 30	
14	TRINITY_DN1	3.85	2e−08	1e−06	30 x 1	
15	TRINITY_DN1	0.04	2e−08	1e−06	1 x 27	
16	TRINITY_DN1	0.69	2e−08	3e−06	30 x 4	
17	TRINITY_DN1	0.08	5e−08	3e−06	22 x 18	
18	TRINITY_DN2	0.96	6e−08	3e−06	30 x 3	
19	TRINITY_DN8	0	9e−08	3e−06	18 x 14	
20	TRINITY_DN1	1.46	1e−07	3e−06	30 x 29	

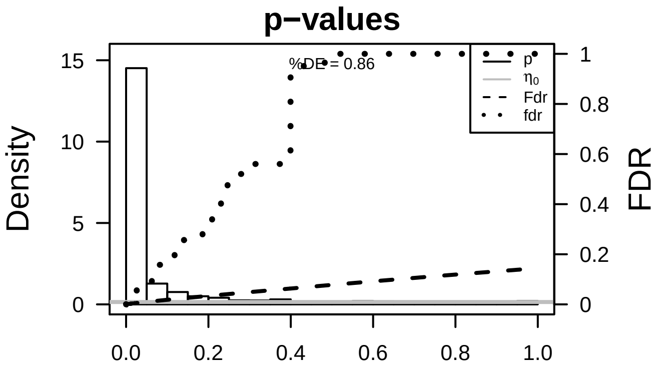

Supplement: Supplementary file 1 [file cells-09-00779-s001.zip › Supplementary materials/FileS4.zip!/FileS3/TFs - Results/Summary Sheets - Differences/Reports/Zhewan.1.25..vs.Zhongwan.6.10..pdf]
